# Effects of Selenium on Morphological Changes in *Candida utilis* ATCC 9950 Yeast Cells

**DOI:** 10.1007/s12011-015-0415-3

**Published:** 2015-07-14

**Authors:** Marek Kieliszek, Stanisław Błażejak, Anna Bzducha-Wróbel, Agnieszka Kurcz

**Affiliations:** Department of Biotechnology, Microbiology and Food Evaluation, Faculty of Food Sciences, Warsaw University of Life Sciences—SGGW, Nowoursynowska 159 C, 02-776 Warsaw, Poland

**Keywords:** Selenium, Morphology, *Candida utilis*, Yeast cells

## Abstract

This paper presents the results of microscopic examinations of the yeast cells cultured in yeast extract–peptone–dextrose (YPD) media supplemented with sodium selenite(IV). The analysis of the morphological changes in yeast cells aimed to determine whether the selected selenium doses and culturing time may affect this element accumulation in yeast cell structures in a form of inorganic or organic compounds, as a result of detoxification processes. The range of characteristic morphological changes in yeasts cultivated in experimental media with sodium selenite(IV) was observed, including cell shrinkage and cytoplasm thickening of the changes within vacuole structure. The processes of vacuole disintegration were observed in aging yeast cells in culturing medium, which may indicate the presence of so-called ghost cells lacking intracellular organelles The changes occurring in the morphology of yeasts cultured in media supplemented with sodium selenite were typical for stationary phase of yeast growth. From detailed microscopic observations, larger surface area of the cell (6.03 μm^2^) and yeast vacuole (2.17 μm^2^) were noticed after 24-h culturing in the medium with selenium of 20 mg Se^4+^/L. The coefficient of shape of the yeast cells cultured in media enriched with sodium selenite as well as in the control YPD medium ranged from 1.02 to 1.22. Elongation of cultivation time (up to 48 and 72 h) in the media supplemented with sodium selenite caused a reduction in the surface area of the yeast cell and vacuole due to detoxification processes.

## Introduction

Selenium is a basic element, the trace amounts of which are essential for the functioning of numerous living organisms [1]. Due to the small differences between deficiency and toxic selenium excess, high availability of this element may negatively affect the cells. Among all known compounds of selenium, sodium selenite(IV) exhibits the highest toxicity [2, 3].

The mechanisms of toxic effects of selenium on yeast cells remain poorly understood. The inhibitory effect of this element may be due to selenium introduction or binding by yeast cell proteins in which it replaces sulfur, primarily substituting it for amino acids such as cysteine and methionine. This process can lead to changes in the tertiary structure of proteins and to enzymes dysfunction. Toxic activity of inorganic selenium compounds in the cells involves reaction of selenites (IV) with thiol groups. At high concentrations, it may result to toxic activity in oxidation processes and a disruption in alkylation processes of catecholamines may occur [4].

Little is known about the selenium transport into the cells of yeast, which is the first step in the process of this element metabolism. The processes of reduction, methylation, and mechanisms for selenium-containing amino acids formation are significant in intracellular selenium transformations. Due to chemical similarity of sulfur and selenium [5], microorganisms absorb selenium into the cytosol, using enzymatic carriers such as sulfate permeases Sul1 and Sul2 [6]. Moreover, the studies on the presence of other transport systems, specific for selenium uptake [7], applying the system of phosphates or monocarboxylic acids transport, were performed [8, 9]. Mapelli et al. [10] reported that an effectiveness of selenites (IV) uptake was reduced in the presence of sulfate(IV) ions in the culture environment. The processes of transformation of inorganic forms of sodium selenite into its organic compounds or production of selenium in the elemental form observed in yeast cells are examples of detoxification mechanisms allowing the survival of microorganisms in a culture medium containing elevated concentration of this element.

In the late 1980s, genotoxic effects in yeast cells were observed when the occurrence of various genetic effects resulting from yeast exposure to sodium selenite(IV) were analyzed. These compounds exhibited toxic, mutagenic activity and influenced yeast genetic recombination [11]. The study presented by Letavayová et al. [12] demonstrated that sodium selenite(IV) is toxic due to its abilities leading to formation of reactive oxygen species and oxidation of thiol groups. The data presented also suggest that during induction of oxidative DNA damage, sodium selenite may generate double-stranded DNA chain breaks in the process of replication. Resulting changes may consequently lead to formation of chromosomal aberrations or apoptosis in yeast cells. These processes may affect the morphological changes in yeast cells, as evidenced by an occurrence of disruptions in membrane channels and functioning of transporters [12–14].

Under high concentration of selenium, yeasts store this element in the cell vacuoles similarly as they do in the presence of heavy metals. The transport of selenium to the yeast cell interior is carried out using glutathione through a transporter belonging to the ABC family (protein Ycf1p) located in the vacuolar membrane. Addition products of selenium are formed during the mentioned process. Separation of selenite ions protects the cells from the toxic effects. However, this mechanism, despite an efficient transfer of selenium in the form of selenodiglutathione (GS–Se–SG) into the vacuole, does not contribute to reduced toxicity. According to Lazard et al. [8], selenium present inside the yeast cell reacts with thiol groups of glutathione leading to the formation of selenodiglutathione and oxidization of glutathione. GS–Se–SG is then reduced via three pathways: (a) by GSH, (b) as a result of enzymatic processes by glutathione, and (c) thioredoxin reductase to GS–Se–H, and finally to volatile hydrogen selenide, which penetrating vacuole membrane is returned to the cytosol posing a threat for the whole yeast cell. High concentrations of GSSG in yeast cells may be also dangerous due to the possibility of this compound reacting with protein thiol groups. The products obtained from these reactions are glutathione–protein disulfides [9].

Detoxification processes occurring in the yeast cells can induce conformational changes in proteins, which consequently leads to their functional impairment. The occurrence of the red coloration of cell biomass indicates the formation of elemental selenium in yeast cells, which is an example of metabolic processes responsible for the reduction of selenium toxicity in yeast cells. The consequences of these processes are the morphological changes in yeast cells.

The size and shape of the yeasts can be modified by environmental factors that may cause the morphological changes in cells [15]. Cells of microorganisms have to maintain homeostasis between the culturing environment and the cell cytosol, which in turn affects the adaptation of cells to changing environmental conditions. The aim of this study was to determine the effects of applied selenium dose in the experimental medium on the morphology of *Candida utilis* ATCC 9950 yeast cells. The results obtained allow expansion of the knowledge concerning an ability of adaptation of yeasts to the environmental conditions rich in selenite ions. This could be related to the possibility of identification of accumulation of different selenium compounds by yeast cell structures depending on the occurrence of the changes in yeast cell structure.

## Materials and Methods

### Biological Material

The study involved the strain of fodder yeasts *C*. *utilis* ATCC 9950 originated from pure culture collection from the Division of Food Biotechnology and Microbiology, Warsaw University of Life Sciences (SGGW). The used strain was maintained in a laboratory on yeast extract–peptone–dextrose (YPD) agar medium at 4 °C.

### Microbiological Media

Liquid YPD medium enriched in sodium selenite (Na_2_SeO_3_) was used as an experimental medium for submerged yeast cultures. Active acidity level of the medium was found to be 5.0. Media and aqueous solution of sodium selenite were sterilized at a temperature of 121 °C for 20 min. The working salt solution was then added to sterile YPD media in such volumes, so that the final selenium content in the experimental media was 20, 30, or 40 mg/L.

### Yeast Cultures

The cultures were maintained in 500-mL spherical flat-bottom flasks containing 90 mL of liquid control or experimental medium. A 10 % volume of cell suspension proliferated in a culture inoculation (4.0–5.0 × 10^8^ cfu/mL) was used for media inoculation. The cultures were maintained on a reciprocating shaker (SM-30 Control E. Büchler, Germany), with an amplitude of vibrations of 200 cycles/min, at a temperature of 28 °C for 72 h.

### Morphological Measurements of Yeast Cells

For microscopic examination, yeast cells were collected from the 24-, 48-, and 72-h cultures in control and experimental media. Microscopic observations were carried out using an immersion lens with a camera fixed on the MB300 light microscope (OPTA-TECH, Poland). The measurements of the cross-sectional area of the cells and vacuoles, the width, and the length of yeast cells were recorded using the computer software OptaView 7 (Poland). Morphological evaluation included 100 yeast cells. The obtained images were digitally processed using Adobe Photoshop CS3 software (Adobe Systems).

### Yeasts Observations Under a Transmission Electron Microscope

The biomass of centrifuged yeast cells (3000×*g*, 10 min, 4 °C) was fixed in 2.5 % glutaraldehyde for 2 h at a temperature of 4 °C. After fixation, the sample was rinsed with 0.025 M phosphate buffer of pH 7.2 for 2 h at a temperature of 4 °C. Final fixing was conducted in a solution of 1 % osmium tetroxide for 1 h at a temperature of 4 °C. Biomass of yeast cells was dehydrated in an increasing gradient of ethylene and acetone supersaturation. The dehydrated yeast cells were immersed in epoxy resin (EPON 812). Immersed material was placed for 24 h at room temperature and then transferred to 60 °C where it was kept for 48 h. The resin blocks with immersed material were cut using a diamond knife on ultra-thin specimens using an ultramicrotome (LBK Ultramicrotome, Sweden) and were mounted on copper grids. Each grid was contrasted in 9 % uranyl acetate and 0.5 % lead citrate. Prepared specimens were observed under a transmission electron microscope JEM 1220 TEM (JEOL, Japan).

### Statistical Analysis

The results obtained were subject to analysis of variance using Statgraphics Plus 5.1 software. Significance of the differences between mean values in particular groups was verified using Tukey’s test at significance level *α* = 0.05.

## Results and Discussion

The presence of different elements in the culturing media can lead to morphological changes in the cultured microorganisms [16]. According to the literature data [17–19], selenium presence in culturing environment affects the changes in the size of cells and vacuole of the yeasts as a result of adaptation processes of cells to changing environment conditions.

**Fig. 1:**
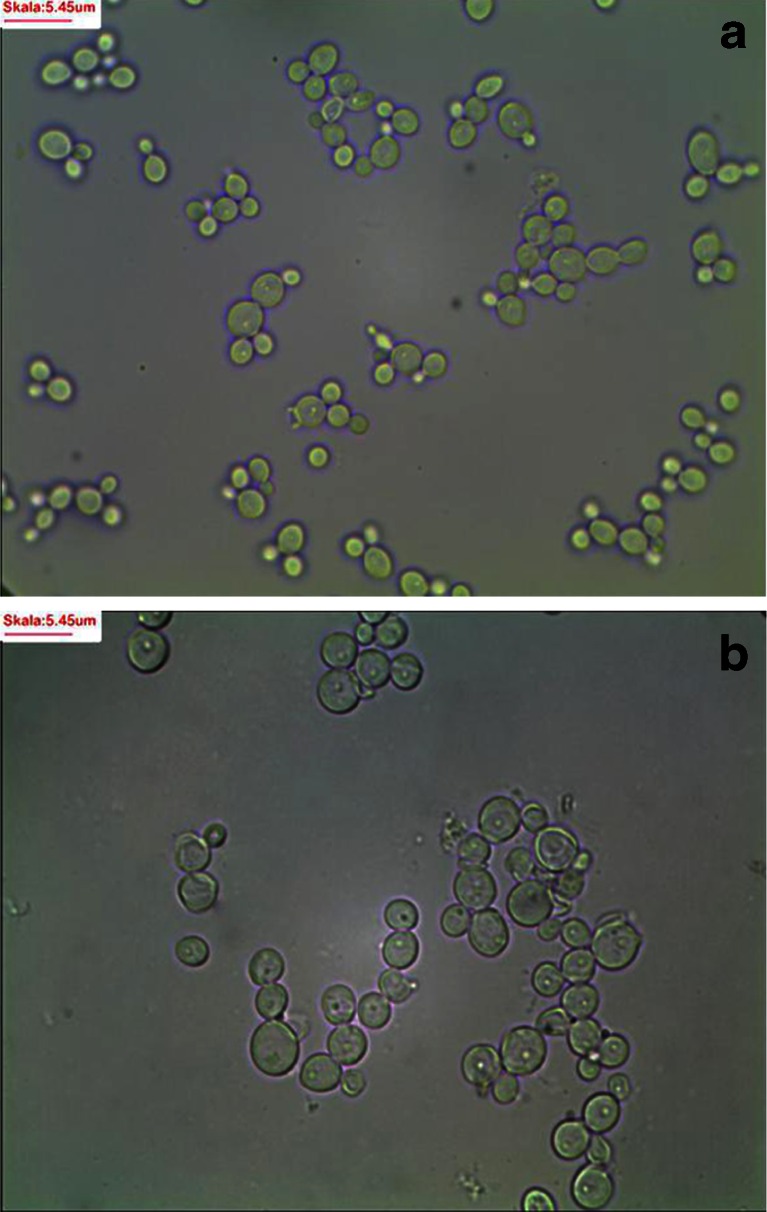
Exemplary microscopic photographs of *C*. *utilis*. **a** YPD control and**b** experimental medium enriched with 30 mg Se^4+^/L

Analysis of microscopic images of the yeasts demonstrated that selenium presence in experimental media (20, 30, and 40 mg Se^4+^/L) caused a significant increase in cross-sectional area of yeast cells and vacuoles after 24-h culture compared to the cells cultured in the control YPD medium (Fig. 1, Table 1). The study conducted by Gharieb and Gadd [19] on *Saccharomyces cerevisiae* yeasts demonstrated that selenium accumulation was mainly observed in vacuoles. Electrochemical potential of vacuole membrane enhanced transport of cations and other substances to these organelle interior.

**Table 1:** Morphological changes of *C*. *utilis* yeast cells (cell and vacuole cross-sectional areas) during culturing in the control YPD medium and experimental media enriched in selenium salts (Na_2_SeO_3_)

Selenium content in the medium (mg/L)	Culturing time (h)	Cell cross-sectional area *Pk* (μm^2^)	Vacuole cross-sectional area *Pw* (μm^2^)	*Pw*/*Pk* ratio	Shape coefficient
		Mean value ± SD			
0	24	2.50 ± 0.81^a^	0.58 ± 0.26^a^	0.23	1.14
	48	2.92 ± 0.89^ab^	0.75 ± 0.28^a^	0.25	1.20
	72	3.16 ± 0.82^b^	0.89 ± 0.40^ab^	0.28	1.05
20	24	6.03 ± 1.59^f^	2.17 ± 0.95^h^	0.35	1.02
	48	4.79 ± 1.71^cde^	1.73 ± 0.86^ef^	0.36	1.04
	72	4.34 ± 1.54^cd^	1.59 ± 0.75^def^	0.36	1.10
30	24	5.75 ± 1.62^f^	2.06 ± 0.94^gh^	0.35	1.10
	48	5.38 ± 2.04^ef^	1.81 ± 0.88^fg^	0.33	1.13
	72	4.85 ± 1.70^de^	1.85 ± 0.86^fgh^	0.38	1.09
40	24	4.29 ± 1.19^cd^	1.46 ± 0.69^cde^	0.34	1.14
	48	4.15 ± 1.37^c^	1.26 ± 0.55^cd^	0.30	1.14
	72	3.46 ± 0.90^b^	1.15 ± 0.48^bc^	0.33	1.22

*SD* standard deviation

^a-h^Means with the same letter did not differ significantly

Mean cross-sectional area of yeasts obtained from the control medium YPD after 24-h culture was 2.50 μm^2^ and was significantly lower compared to the area of cells obtained from the experimental media supplemented with selenium at a dose of 20 mg Se^4+^/L (6.03 μm^2^). Higher selenium content (30 and 40 mg Se^4+^/L) in culturing media also caused a significant increase in size of the yeast cells (5.75 and 4.27 μm^2^, respectively) compared to the cells cultured in the control YPD medium.

After 48 h of cultivation, the area of the yeast cells from experimental cultures was still significantly larger compared to the cells from the control culture. Mean cross-sectional area of the cells from the control culture was 2.92 μm^2^, while in case of media supplemented with selenium at doses of 20 and 30 mg Se^4+^/L, these values were 4.79 and 5.38 μm^2^, respectively. In case of experimental medium supplemented with selenium at a dose of 40 mg Se^4+^/L, the mean area of yeast cells was 4.15 μm^2^. Further growth in the size of cells (from 2.50 to 3.16 μm^2^) and vacuoles (from 0.58 to 0.89 μm^2^) was observed in the control medium with an elongation of the time of *C*. *utilis* yeasts cultivation (up to 72 h). The reverse tendency was observed in case of cultures maintained in the experimental media supplemented with sodium selenite(IV). Gradual decrease in cross-sectional area of the cells and vacuoles was observed on the second day of culturing, compared to the yeasts obtained from the 24-h culture. According to the statistical analysis, the concentrations of 30 and 40 mg Se^4+^/L in experimental media did not cause any significant changes in surface area of vacuoles in all controlled time intervals. A reduction in the size of surface area of vacuoles was observed during the 72-h submerged culture of *C*. *utilis* yeasts in experimental media supplemented with 20 and 30 mg Se^4+^/L, and this reduction was about 28 and 16 %, respectively, compared to the yeast cells obtained from the 24-h culture. Based on the literature data [20, 21], it may be supposed that an increased size of vacuoles of the yeast cells from the media supplemented with sodium selenite during logarithmic growth phase was caused by selenium accumulation in these organelles. Gharieb and Gadd [19] and Uden et al. [20] reported that gradual vacuole size reduction in the phase of stationary growth was related to mobilization of the process of selenium detoxification in yeast cell structures. This mechanism involves the progressing process of oxidizing inorganic form of selenium, which is more toxic than volatile organic forms of this element. Other detoxification mechanism involves reduction of inorganic selenium bonds to red brown form of elemental selenium.

According to Lazard et al. [8], only a small part of selenium present in the vacuole would have been transported to the cytoplasm using reduced glutathione, and then outside the yeast cell to culturing environment. This process involves low-molecular polyphosphates and transport proteins [22, 23]. The data presented by Zhang et al. [24] suggests that under oxygen-limited conditions, yeast cells have the ability of removing excessive selenium ions from the cell using characteristic transport vesicles formed probably by Golgi apparatus [25].

The coefficient of shape of the yeast cells cultured both in experimental media enriched with sodium selenite and in the control YPD medium ranged from 1.02 to 1.22. Coefficient value close to 1 determines the spherical shape of the cells [26]. An observation after 48- and 72-h culture in media supplemented with selenium at doses of 30 and 40 mg Se^4+^/L led to interesting conclusions of disrupted yeast cells with cell membrane fragments, cytosol, and cell organelle leakage to extracellular environment. This was caused by the weakening of cell membrane structures and an accumulation of metabolism products by yeast cells [27].

**Fig. 2:**
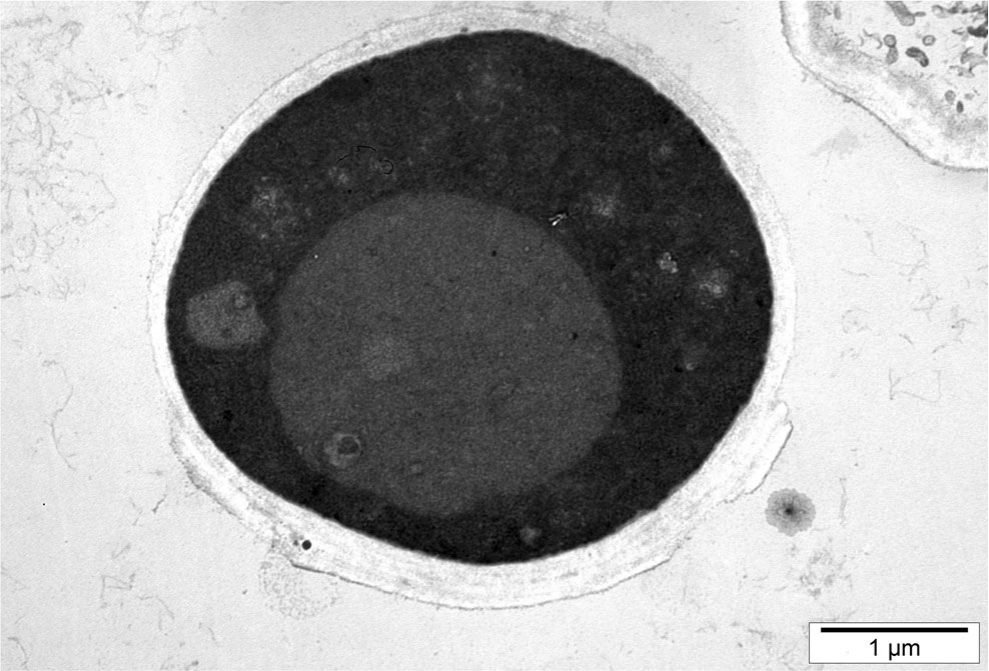
Image of *C*. *utilis* yeast cells from 48-h culture in experimental medium enriched with 20 mg Se^4+^/L (Uneven surface of yeast cell wall)

**Fig. 3:**
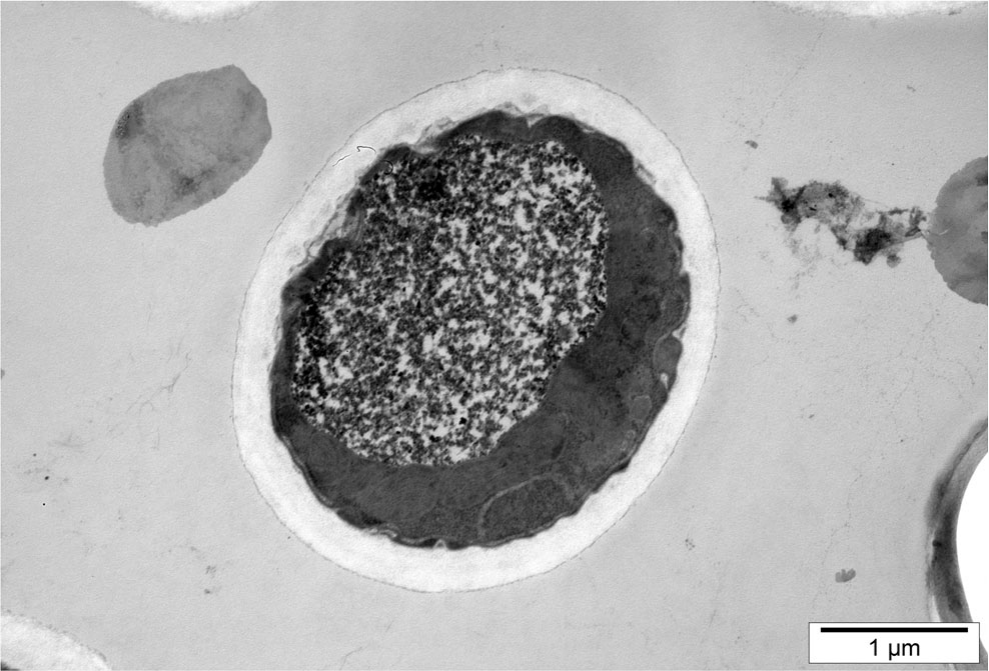
Image of *C*. *utilis* yeast cells from 72-h culture in experimental medium enriched with 30 mg Se^4+^/L (Presence of various metabolic substances inside vacuole)

Yeast cells from experimental cultures containing selenium were large, sometimes elongated, and demonstrated uneven surface of cell membrane (Fig. 2). This resulted from adaptation of cells to new environmental conditions. During the 72-h yeast culture in the medium with selenium at doses of 20, 30, and 40 mg Se^4+^/L, the presence of metabolic substances was observed inside the vacuole (Fig. 3). According to the literature data [28, 29], these probably are low-molecular polyphosphate granules. Gharieb and Gadd [19] reported that *S*. *cerevisiae* yeasts cultured in the medium supplemented with selenium at concentration of 5 mM resulted in an accumulation of the reduction products of selenium, so-called solid particles (inter alia, elemental selenium, Se^0^) inside the vacuoles.

The study conducted by Gerrard et al. [30] demonstrated that *Escherichia coli* was able to reduce sodium selenite(IV) to elemental form during the growth in a culturing environment containing inorganic selenium sources. An occurrence of red coloration of yeast biomass indicates the large content of inorganic selenium in yeast cells. High content of selenium in the cells may result from the deposited free, amorphous selenium.

**Fig. 4:**
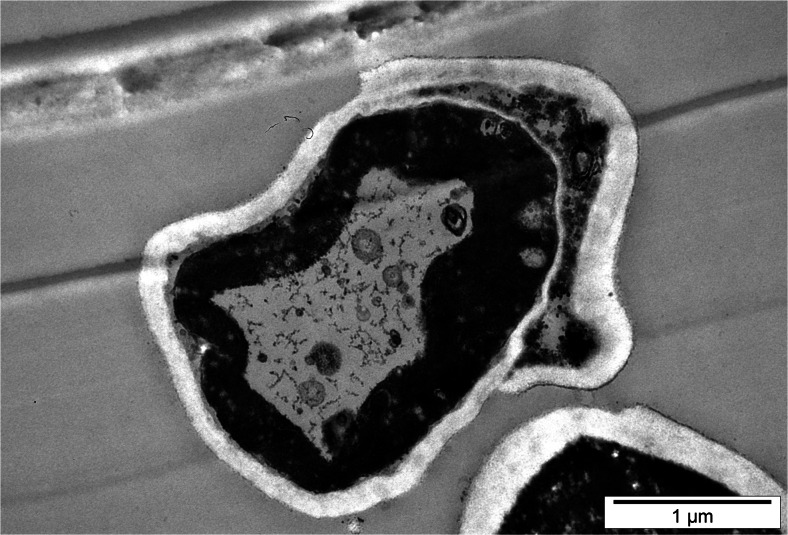
Image of *C*. *utilis* yeast cells from 48-h culture in experimental medium enriched with 20 mg Se^4+^/L (Cell wall folding)

In the presence of selenite ions in experimental medium, and with increasing time of culturing, the cells of *C*. *utilis* yeasts were subject to shrinking, and these changes resulted in “wrinkled cell membranes” (Fig. 4). Yeast cells reduced the pressure inside the cell, and thus the cell membranes that were not subject to damage become more wrinkled as a result of formation of cell furrows and folds. The consequence of these processes was a reduction in cell size.

**Fig. 5:**
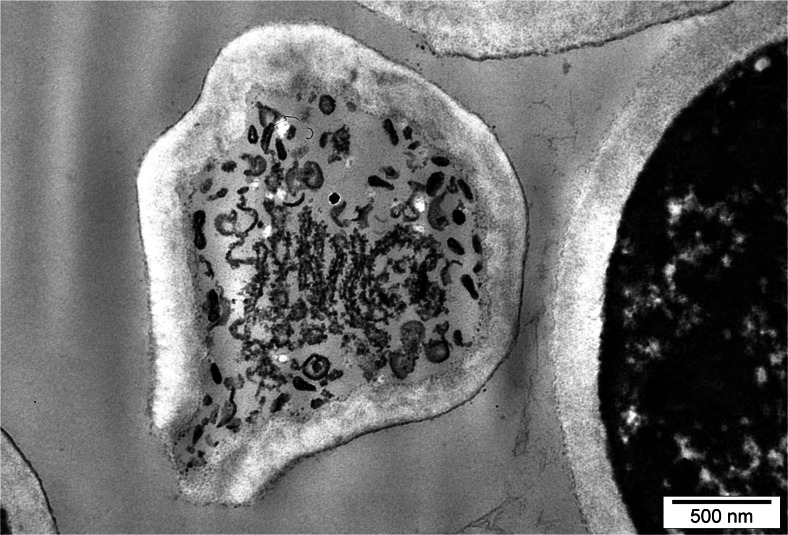
Image of *C*. *utilis* yeast cells from 72-h culture in experimental medium enriched with 40 mg Se^4+^/L (Cell wall breaking and cytoplasm leakage from the cell, degradation of intracellular structures)

**Fig. 6:**
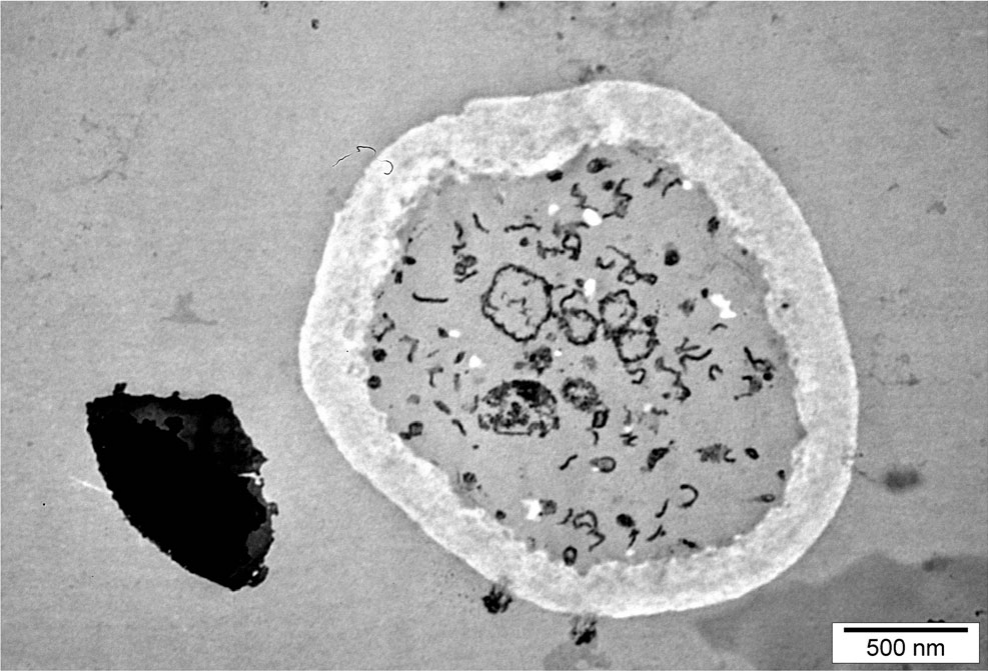
Image of the ghost cells of *C*. *utilis* yeast cells from 72-h culture in experimental medium enriched with 30 mg Se^4+^/L (ghost cells of the yeasts lacking intracellular organelles)

After 48- and 72-h culture in experimental media supplemented with sodium selenite, an analysis of an image of some cells of *C*. *utilis* demonstrated that the yeast cells contained granularities without clearly marked intracellular structures. In an initial stage of autolysis, it was possible to observe the periplasmic space, pyknosis processes, and cytoplasmic vacuolization in the yeast cells (Fig. 5) as well as fragmentation of the cell nucleus (karyorrhexis) [31]. Moreover, degradation of intracellular structures was observed in aging yeast cells, which consequently led to cell membrane breaking and cytoplasm leakage from the cells (Fig. 5). The observed so-called ghost cells are a result of these processes (Fig. 6).

According to Martínez-Rodríguez et al. [32], progressing process of yeast cell autolysis results in a decreased volume of the cells, which leads to the leakage of cytoplasmic material being an effect of wall–membrane complex of yeast cell damage.

Based on microscopic observations, it was observed that an elongation of the time of yeast culturing (up to 72 h), and increased sodium selenite doses in experimental media, unprofitably affected the cells of *C*. *utilis* ATCC 9950 yeasts. Observed changes in cross-sectional area of the cells after 48- and 72-h culture would have been a result of disturbances in yeasts functioning and metabolism caused by sodium selenite addition to experimental media.

The study presented by Rajashree and Muthukumar [18] demonstrated that *S*. *cerevisiae* NCYC 1026 yeasts cultured in Sabouraud medium (SDB) enriched with sodium selenite at various doses exhibited distinct metabolic changes. The surface of yeast cells cultured in the control medium was smooth, while it was rough in the medium supplemented with selenium at a dose of 50 mg/L. In case of yeasts culturing in a medium with 150 mg/L addition, the cells were deformed.

The differences in the size of *C*. *utilis* ATCC 9950 yeasts in the media with sodium selenite compared to the cultures in the control medium YPD resulted from this element accumulation in cell organelles and vacuoles. It may be supposed that high selenium concentrations in experimental medium caused damages in cell membrane and cytoplasmic membrane of the examined *C*. *utilis* strain. The study of Dilsiz et al. [33] suggests that selenium affected the composition of cytoplasmic membrane of *S*. *cerevisiae* yeasts. This phenomenon was related to an increase in saturated fatty acid content and concurrent reduction of unsaturated acids in cytoplasmic membrane. This was probably related to selenium incorporation in the site of sulfur in amino acids [23, 34]. Similar hypothesis was presented by Letavayová et al. [35], who observed that selenium incorporates into free sulfhydryl groups (–SH) of enzymatic proteins. The consequence of these changes is the formation of selenium thiosulfates (S–Se–S) [35], which change protein structure, and in the case of enzymatic proteins, their catalytic activity [36]. Modified proteins affect the changes in metabolism of yeast cells, which would have been reflected in observed morphological changes of *C*. *utilis* ATCC 9950 yeasts obtained from the sodium selenite-enriched experimental media.

## Conclusion

The results obtained show that the presence of sodium selenite in an experimental medium led to morphological changes in the cells of *C*. *utilis* ATCC 9950 yeasts. It is commonly known that selenium generates oxidation stress inside the yeast cell, which damages the structures like DNA, proteins, and other important macro-components. An example of observed morphological changes was an increase in the size of the cells, shrinkage of the yeasts, cytoplasm thickening, or the change in vacuole structure. Aforementioned observations pointed to some disruptions in metabolic activity and structural stability of the examined cells of *C*. *utilis* yeasts. The course of selenium metabolism in yeast cells is a very complex mechanism, which is recognized only partially. It is commonly believed that one of such processes was the selenium detoxification in yeast cells. Metabolic changes observed in the yeasts did not affect the differentiations in coefficient shape of the yeast cells.
